# Root type matters: measurement of water uptake by seminal, crown, and lateral roots in maize

**DOI:** 10.1093/jxb/erx439

**Published:** 2018-01-03

**Authors:** Mutez Ali Ahmed, Mohsen Zarebanadkouki, Félicien Meunier, Mathieu Javaux, Anders Kaestner, Andrea Carminati

**Affiliations:** 1 Division of Soil Hydrology, University of Goettingen, Göttingen, Germany; 2 Chair of Soil Physics, University of Bayreuth, Bayreuth, Germany; 3 Earth and Life Institute, Université catholique de Louvain, Louvain-la-Neuve, Belgium; 4 Laboratory for Neutron Scattering and Imaging, Paul Scherrer Institute, Villigen, Switzerland

**Keywords:** Crown roots, diffusion–convection model, neutron radiography, root water uptake, seminal roots, *Zea mays*

## Abstract

The ability of plants to take up water from the soil depends on both the root architecture and the distribution and evolution of the hydraulic conductivities among root types and along the root length. The mature maize (*Zea mays* L.) root system is composed of primary, seminal, and crown roots together with their respective laterals. Our understanding of root water uptake of maize is largely based on measurements of primary and seminal roots. Crown roots might have a different ability to extract water from the soil, but their hydraulic function remains unknown. The aim of this study was to measure the location of water uptake in mature maize and investigate differences between seminal, crown, and lateral roots. Neutron radiography and injections of deuterated water were used to visualize the root architecture and water transport in 5-week-old maize root systems. Water was mainly taken up by crown roots. Seminal roots and their laterals, which were the main location of water uptake in younger plants, made a minor contribution to water uptake. In contrast to younger seminal roots, crown roots were also able to take up water from their most distal segments. The greater uptake of crown roots compared with seminal roots is explained by their higher axial conductivity in the proximal parts and by the fact that they are connected to the shoot above the seminal roots, which favors the propagation of xylem tension along the crown roots. The deeper water uptake of crown roots is explained by their shorter and fewer laterals, which decreases the dissipation of water potential along the roots.

## Introduction

In view of the increasing demand for food by the growing population and of the limiting water availability in many countries, there is an urgent need to understand what root properties facilitate the extraction of water from drying soils (Bishopp and Lynch, 2015). Maize (*Zea mays* L.) is one of the most important food crops worldwide. Despite its importance, there is limited information on the function of the different root types in extracting water from the soil. The maize root stock has a unique architecture that comprises several root types forming at different developmental stages (Hochholdinger *et al.*, 2004). The embryonic primary and seminal roots together with their laterals constitute the major portion of the seedling root stock during the first 2 weeks after germination. Later in development, the post-embryonic shoot-borne roots (i.e. crown roots) become the major backbone of the maize root system (Hochholdinger, 2009).

The literature on the hydraulic properties of maize roots is largely dominated by measurements of primary and seminal roots mainly growing in aeroponic systems (Frensch and Steudle, 1989; Varney and Canny, 1993; Steudle, 2000). For instance, McCully and co-workers found that the distal parts of seminal roots have immature, not conductive, xylem and they do not take up water (Wang *et al.*, 1991), and such conclusions have been generalized to all maize root types. Similarly, prominent models of water uptake by maize roots assumed that seminal and crown roots have identical hydraulic properties (Doussan *et al.*, 1998; Leitner *et al.*, 2014; Robbins and Dinneny, 2015). However, is it safe to extend the results of seminal roots to crown roots? Also, in general, do different root types contribute equally to root water uptake?

Recently, we showed that for 2-week-old maize plants, water was mainly taken up by laterals roots and the function of the primary and seminal roots was to transport water to the shoot (Ahmed *et al.*, 2016). We expected that these results cannot be easily generalized to mature maize, because a fundamental part of the root system (crown roots) has not yet formed at this stage. The objective of this study was to measure root water uptake by a mature maize root system growing in soil and investigate differences between seminal, crown, and lateral roots.

We used neutron radiography to image the spatial distribution of maize roots in soil and trace the transport of injected deuterated water (D_2_O) in soil and roots just as in Ahmed *et al.* (2016). The transport of D_2_O was simulated using a diffusion–convection numerical model. By fitting the observed D_2_O transport, we quantified the diffusion coefficient and the water uptake of the different root segments. The method was developed and successively tested with lupines (Zarebanadkouki *et al.*, 2014) and 2-week-old maize (Ahmed *et al.*, 2016). Here, we applied this method to measure root water uptake by a mature maize root system. The measurements of the fluxes were complemented with measurements of the axial hydraulic conductivities of seminal and crown roots at different positions.

## Materials and methods

### Soil and plant preparation

Sandy soil was collected from the artificial catchment of Chicken Creek located near Cottbus, Germany, and it was left to dry at ambient humidity for 3 d. Twelve slabs (40 cm wide and 38 cm high) were laid horizontally and nine plastic bars (1 cm thick) were placed inside containers, forming a grid and dividing the internal space into 16 compartments (for more details, see Ahmed *et al.* (2016)). The soil was poured into each compartment through a 2 mm sieve and then wetted. The grids were then gently removed from the containers and the space between the compartments was filled with coarse sand that hydraulically disconnected the compartments. The top sheet of each container was then closed and the containers were gently turned vertically. The detachable front sheet of the containers had holes of 1 mm diameter at regular intervals, through which D_2_O was injected.

Maize seeds were germinated on moist filter paper for 48 h and then planted in the containers. The upper soil layer was covered with quartz gravel to reduce evaporation. The plants were grown with a daily light cycle of 14 h and 10 h of darkness, light intensity of 500 µmol m^−2^ s^−1^, day:night temperature of 24:19 °C, and relative humidity of 60%. The plants were 5 weeks old when the neutron radiography measurements started. Throughout the whole period, the soil water content was maintained between 0.10 cm^3^ cm^−3^ and 0.15 cm^3^ cm^−3^.

### Neutron radiography

Neutron imaging was used to image water and root distribution in soil thanks to its high sensitivity to hydrous materials (Ahmed *et al.*, 2016). A parallel neutron beam propagates through the sample and the transmitted neutrons behind the sample are detected using a scintillator. The scintillator converts the neutrons into visible light acquired using a CCD camera. The detected image carries the information on the thickness and composition of the sample according to the Beer–Lamberts law.

The experiments were performed at the ICON beam-line of the Paul Scherrer Institute (PSI), Switzerland. We used a camera with an array of 2160 × 2560 pixels, resulting in a field of view of 13.3 × 16 cm and pixel size of 0.062 mm. A lamp identical to those in the growth chamber was mounted above the samples during the radiography in the daytime. Plants were kept in the imaging station for 1 h before the experiments with deuterated water began.

### Deuterated water

Water flow into roots was traced using D_2_O. D_2_O has a lower neutron attenuation coefficient than H_2_O and this contrast makes it is easily visible in the radiographs. D_2_O has long been used in the study of water flow in plants because it behaves similarly to water (Ordin and Kramer, 1956; Da Ines *et al.*, 2010; Warren *et al.*, 2013). A volume of 10–15 ml of D_2_O was locally injected into selected soil compartments using a syringe, and its transport was imaged with a time resolution of 20 s per picture for 2–3 h.

### Image analysis

Detailed information on the image processing can be found in Ahmed *et al.* (2016). Briefly, neutron radiographs were referenced to a flat field (radiograph without sample) and dark current (signal recorded by the camera in the absence of a beam). One of our container frames remained unplanted to determine the neutron attenuations of aluminum and dry soil. After subtracting the contribution of aluminum and dry soil, the remaining values represented the water content in the sample. Because there was a clear contrast between the roots and the surrounding soil, we were able to distinguish and segment the roots from the soil.

The concentrations of H_2_O and D_2_O were estimated assuming that there was a rapid re-equilibration of the liquid pressure upon irrigation. In other words, we assumed that the liquid content (H_2_O plus D_2_O) was constant shortly after injection. Note that the samples were kept under moist conditions. The signal in the pixels containing the roots comprised the attenuation coefficients of the roots and of the soil in front of and behind the roots in the beam direction (across soil thickness). We calculated the actual contributions of H_2_O and D_2_O in the roots assuming that the amounts of H_2_O and D_2_O in the soil in front of and behind the roots were equal to those of the soil at the sides of the roots. The volumetric concentration of D_2_O in roots (C_r_) and soil (C_0_) was calculated as the thickness of D_2_O divided by the total liquid thickness in roots and soil, respectively. C_r_ and C_0_ were averaged along the segment of roots immersed in D_2_O.

### Model of D_2_O transport into roots

The time-series neutron radiographs allow visualization of the transport of D_2_O into the roots. However, a quantitative estimation of root water uptake requires simulation of D_2_O transport. We used a diffusion–convection model of D_2_O transport in roots (Zarebanadkouki *et al.*, 2014; Ahmed *et al.*, 2016). Diffusion is driven by gradients in D_2_O concentration between soil and root. Convection is driven by net water uptake (Ahmed *et al.*, 2016). The region from the root surface to the outer radius of the xylem was treated as a homogeneous flow domain in which water flows radially. We did not distinguish between apoplastic and cell to cell water pathways (the assumption is justified by the sensitivity analysis in Zarebanadkouki *et al.*, 2014). Additionally, we did not explicitly include an endodermis as a region with a distinct diffusion coefficient. The water flow from the lateral roots into the primary, seminal, and crown roots was included in the model as a sink into the xylem of the seminal and crown roots. We described the change in concentration of D_2_O in the roots as:

$$\theta \frac{\partial c^{}}{\partial t}=\frac{\partial }{r\partial r}\left(rD(\frac{\partial c}{\partial r})\right)-\frac{\partial }{r\partial r}\left(rj_{{}_{r}}^{}c^{}\right)-\frac{\partial }{\partial x}\left(j_{x}c\right)+S^{\text{lat}}c_{x}^{\text{lat}}$$ (1)

where *r* is the radial co-ordinate (cm), *x* is the longitudinal coordinate (cm), θ(*r*,*x*) is the water content (cm^3^ cm^−3^) (variable in the soil and equal to 1 in the root), *t* is the time (s), *c*(*r*,*x*,*t*) is the concentration of D_2_O in root (cm^3^ cm^−3^), *D*(*x*) is an effective diffusion coefficient of D_2_O in the root tissue (cm^2^ s^−1^), *j*_*x*_(*r*,*x*) is the axial flux of water (cm s^−1^), *j*_*r*_(*r*,*x*) is the radial flux of water (cm s^−1^), *S*^lat^(*r*,*x*) is a sink term giving the flux of water delivered into the xylem of the axial root from the lateral roots (s^−1^) and $c_{x}^{\text{lat}}$ is the concentration of D_2_O in the xylem of the lateral roots.

When the root segment immersed in D_2_O has no laterals, we imposed *S*^lat^(*r*,*x*)=0. When the root segment immersed in D_2_O comprises lateral roots, *S*^lat^ is given by:

$$\text{for }\{\begin{array}{cc} r\leq r_{x} & S^{\text{lat}}=-\frac{2r^{\text{lat}}L_{}^{\text{lat}}j_{r}^{\text{lat}}}{r_{x}^{2}L_{im}^{}}\\ r>r_{x} & S^{\text{lat}}=0 \end{array}$$ (2)

where *r*^lat^ is the radius of lateral roots, $j_{r}^{\text{lat}}$is the radial flux of water into the lateral roots (calculated at the root surface), *L*^lat^ is the total length of lateral roots connected to the primary/seminal root immersed in D_2_O, *r*_*x*_ is the xylem radius of the primary/seminal root, and *L*_im_ is the length of the primary/seminal root immersed in D_2_O.

We further assumed that the axial transport of water (D_2_O) occurs only in the root xylem. From mass conservation, the axial flux of water in the xylem can be calculated as:

$$\pi r_{x}^{2}\frac{\partial j_{x}\left(x\right)}{\partial x}=2\pi r_{x}j_{r}\left(r_{x},x\right)+\pi r_{x}^{2}S^{\text{lat}}\left(x\right)$$ (3)

### Model implementation

We used our model to fit the changes of D_2_O concentration *C*_root_ along the root segments immersed in D_2_O. The parameters to be fitted were: (i) the diffusion coefficients of the roots *D*; and (ii) the convective fluxes into the roots, *j*_*r*_ and, for branching roots additionally the sink term, *S*^lat^. For the experiments at night, we assumed that the convective fluxes were negligible and the parameter to be fitted was *D*. For the experiments during the day, we assumed that the diffusion coefficient was equal to that of the night experiment and we varied the convective fluxes until we found an optimal match between the observed and simulated *C*_root_ along the root.

For the numerical implementation of the model, we followed the approach described by Ahmed *et al.* (2016). We discretized Equation 1 numerically using the finite-difference method. Each variable was represented as a 2D computational grid with *N*_*r*_ equally spaced grid elements along the root radius (51 nodes) and *N*_*x*_ grid elements along the root length (60 nodes). The time derivatives were discretized with an explicit method. We used a time step of 5 × 10^–2^ s. The inverse problem was solved in matlab suing the ‘fmincon’ solver from its optimization toolbox.

### Measurements of axial hydraulic conductivities

The root pressure probe consists of a pressure transducer and a capillary that connects an excised root system to the probe (Frensch and Steudle, 1989; Liu *et al.*, 2009). Here we used it by imposing a constant pressure difference and measuring the water flow following the approach of Bramley *et al.* (2009). Half of the capillary (diameter of 600 µm) was filled with silicon oil and the other half of the capillary facing the root was filled with water. The meniscus between water and oil was used to measure the water volume injected into and sucked from the root. The probe was equipped with a metal rod that could push or suck water into or out of the root.

Three maize plants were grown in the same conditions, containers, and soil as in the neutron radiography experiments. After 5 weeks, the samples were opened, and seminal and crown roots were excised in water and then connected to the probe. The tightness of the connection between the root and the probe was achieved using a silicon material that was pressed around the root (Xantopren L blue, Heraeus Kulzer GmbH, Germany). The root pressure probe was laid horizontally and the root was placed in a water bath.

Crown and seminal roots that were between 5 cm and 45 cm long were excised under water at different positions and sealed to the root pressure probe. When the root pressure reached a constant value (it took ~30–120 min), the root was further cut at a distance of 2 cm from the end of the silicon seal with a sharp razor blade. In this way, the measured root segment was 2 cm long (including the part in the sealing) and it was open on both sides, so that the flow depended on the axial conductivity. Note that we only measured unbranched segments to simplify the interpretation of the results. A series of constant pressure experiments were carried out to induce water flow into the root (3–5 pressure clamps). Root pressure was increased in 20–50 kPa increments and held constant for 10–120 s by continually adjusting the position of the metal rod inside the pressure probe. The distance that the metal rod had moved was used to calculate the flow rate *Q* (m^3^ s^−1^). The slope of the linear regression of flow rate *Q* plotted against the applied pressure gradient Δ*P*/*dl* (MPa s^−1^), where *dl*=2 cm is the length of the root segment, gives the axial conductivity of the root segment, *K*_*x*_ (m^4^ MPa^−1^ s^−1^). The axial conductivity was determined at varying positions along seminal and crown roots, considering the mid-length of the segment as the measurement position.

## Results

**Fig. 1.**
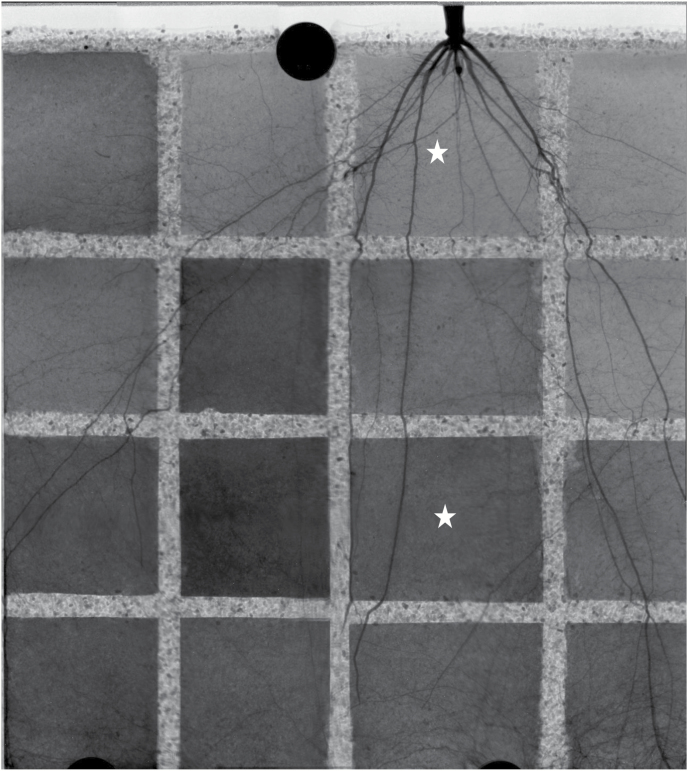
Reconstructed image of one sample before the injection of deuterated water (D_2_O). The figure shows the roots of 5-week-old maize (*Zea mays* L) and the soil water distribution. The gray scales are proportional to the water content. The darker the image, the higher the soil water content. This image was obtained from stitching together 12 radiographs with an original field of view of 13.3 × 16 cm. The stars show two compartments in which we injected D_2_O and monitored its transport into soil and roots.

We monitored the transport of D_2_O into the roots of 12 plants. We injected D_2_O into selected compartments of each sample during the day or night. To illustrate the results, we present the radiographs of one sample in which D_2_O was injected during the daytime into two compartments (Fig. 1). Figure 1 shows the reconstructed image of the sample before D_2_O injection. The image was obtained by overlapping 12 radiographs taken at different locations. The gray values are proportional to the water content: the darker the image, the higher the soil water content. In the radiograph, the roots appear dark due to their high water content. The sharp contrast between roots and the surrounding soil, resulting from the higher volumetric water content in roots, allowed us to segment the root system from the soil. The root architecture of a 5-week-old maize plant consisted of a primary root and seminal roots with long laterals and crown (nodal) roots that emerged from the above-ground part of the plant 2 weeks after planting. Crown roots were thicker than seminal roots and had fewer and shorter laterals (Fig. 1).

**Fig. 2.**
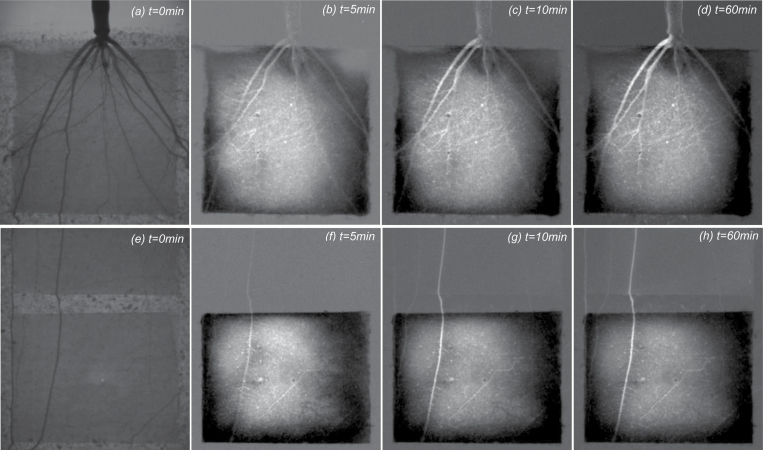
Neutron radiographs of deuterated water (D_2_O) injection in the upper (a–d) and lower (e–h) compartment during the day. (a) The sample before injection (*t*=0). (b–d) The difference between the actual radiograph at time *t* and that before injection (*t*=0). Brighter colors in (b–d) indicate a lower neutron attenuation and higher D_2_O:H_2_O ratio. Conversely, the dark areas show accumulation of H_2_O after D_2_O injection. After injection, the crown roots turned bright immediately, which indicated that D_2_O had entered them faster than the seminal roots and their laterals. (e) The sample before injection. (f–h)The difference between the actual radiograph at time *t* and that before injection (*t*=0). The compartment included the distal parts of a crown root where the laterals had not emerged yet. In contrast to the seminal roots, the crown roots were able to take up water from their most distal part.

Before D_2_O injection, the average soil water content in all compartments of the 12 samples was between 0.10 cm^3^ cm^−3^ and 0.15 cm^3^ cm^−3^, corresponding to soil matric potentials of –70 hPa and –20 hPa, respectively. After injection of D_2_O, the water content increased and the corresponding change in pressure is expected to be ~50 hPa, which is small compared with the difference in water potential between soil and roots. The stars in Fig. 1 show the compartments where D_2_O was injected during the day. The day injection of D_2_O into the upper compartment is presented in Fig. 2. Figure 2a shows the distribution of water and roots in the compartment before D_2_O injection. Proximal segments of seminal and crown roots with their laterals are visible in the compartment. Figure 2b–d shows the difference between the actual radiographs at time *t*, and the radiograph before D_2_O injection (*t*=0). Brighter gray values indicate reduced neutron attenuation as a result of an increased D_2_O:H_2_O ratio. Conversely, the dark areas show accumulation of H_2_O after D_2_O injection. After D_2_O injection, the crown root and their associated lateral roots turned bright much faster than the seminal roots, indicating that the radial transport of D_2_O into the seminal roots and their laterals was significantly slower than that into the crown roots and their laterals. Note that the laterals of seminal roots were the main location for water uptake in 2-week-old maize.

Figure 2e–h shows the injection of D_2_O into a lower compartment during the day. Figure 2e shows the distribution of water and roots in the compartment before D_2_O injection. Distal segments of crown roots with their laterals are visible in the compartment. Figure 2f–h shows the difference between the actual radiographs at time *t*, and the radiograph before D_2_O injection (*t*=0).

After D_2_O injection, the crown root and their laterals turned bright almost immediately. Furthermore, as D_2_O reached the crown roots, it started to move along the roots beyond the capillary barrier (Fig. 2h). In contrast to seminal roots (Ahmed *et al.*, 2016), the crown roots were also able to take up water from their distal segments. We will come back to this point in the Discussion.

**Fig. 3.**
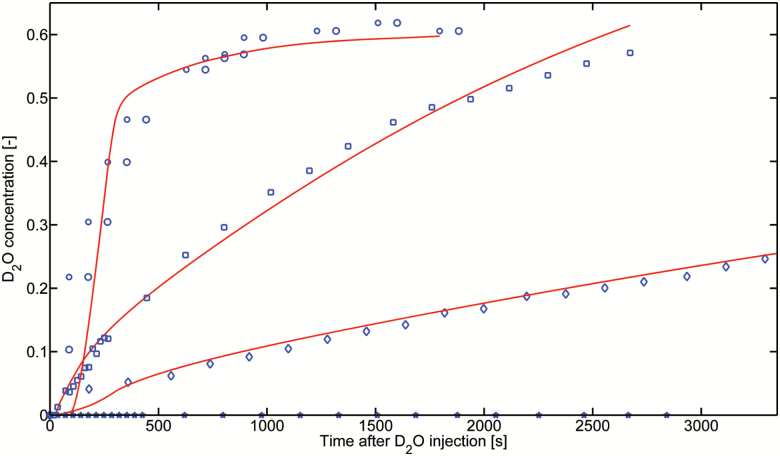
Increase of D_2_O concentration inside the seminal root segments, proximal and distal segments of crown roots, and their laterals measured during the night-time. The concentrations were averaged along the segments immersed in D_2_O. The experiments were simulated, solving numerically the model illustrated in Supplementary Fig. S1 at *JXB* online assuming no convection. The best fits are plotted as solid lines. (This figure is available in color at *JXB* online.)

**Fig. 4.**
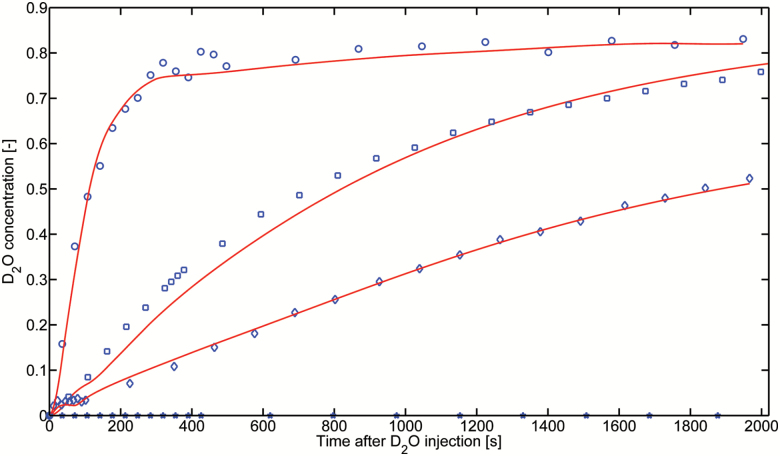
Increase of D_2_O concentration inside the proximal and distal segments of crown and lateral roots during the daytime. The concentrations were averaged along the root segments immersed in D_2_O. The concentrations were fitted assuming that the diffusion coefficient was constant during the day and night and using the convective fluxes as fitting parameters (see text for details). The best fits are plotted as solid lines. (This figure is available in color at *JXB* online.)

The concentration of D_2_O in the root segments immersed in D_2_O was averaged along the root length and root diameter. Figures 3 and 4 show the D_2_O concentration in the proximal segments of the seminal roots, in the distal and proximal segments of the crown roots, and in their laterals (the laterals were mainly at the proximal part of crown roots) during the night and day, respectively. The results show that the D_2_O concentration increased more rapidly in laterals than both the proximal and distal segments of the crown roots. Along the crown roots, D_2_O entered more rapidly in the distal segments than in the proximal segments. D_2_O transport in seminal roots, as well as in their laterals, was very slow, and we did not detect any D_2_O increase in the seminal roots in both compartments, during both the day and night.

**Fig. 5.**
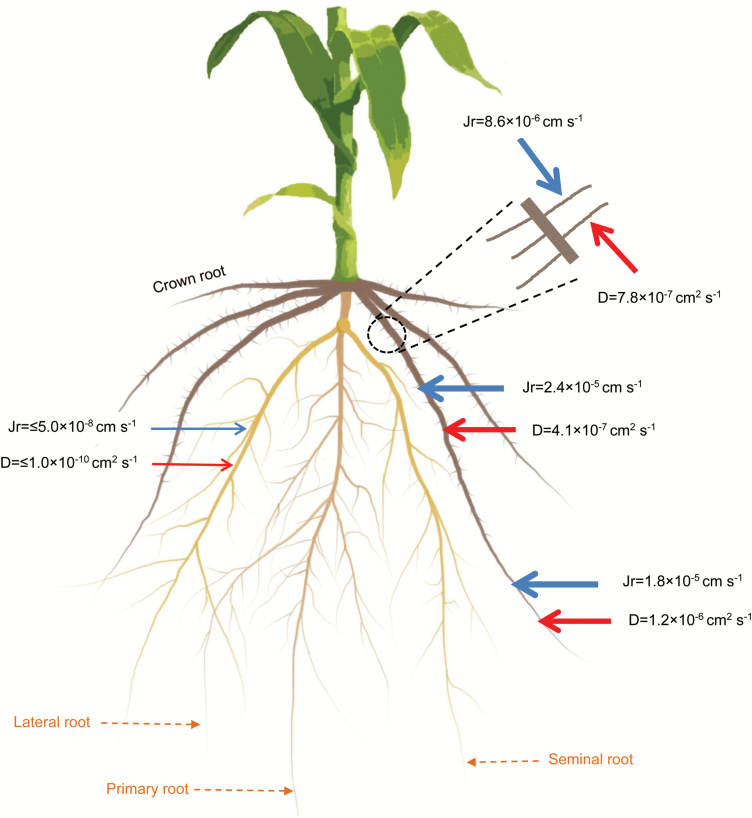
Summary of the results. The figure shows 5-week-old maize with primary, seminal, crown, and lateral roots. The red and blue arrows correspond to the diffusion coefficient and radial fluxes of the different root segments, respectively.

Using our model (Equation 1) to fit the night measurements, we obtained the diffusion coefficient (*D*) of the different root segments. By fitting the day measurements, we then obtained the radial water flow into the roots (*j*_*r*_). The results are summarized in Fig. 5. The diffusion coefficient of seminal roots was below our detection limit (*D*≤1 × 10^−10^ cm^2^ s^−1^) and it was much smaller than the diffusion coefficients of both the proximal (*D*=4.19 ± 0.23 × 10^–7^ cm^2^ s^−1^) and distal (*D*=1.27 ± 0.30 × 10^–6^ cm^2^ s^−1^) segments of crown roots. The diffusion coefficient of the laterals of crown roots was *D*=7.84 ± 0.42 × 10^–7^ cm^2^ s^−1^. Water was mainly taken up by crown roots and their laterals (mostly present on the proximal segments). The radial flow into the distal (*j*_*r*_=1.83 ± 0.15 × 10^–5^ cm s^−1^) and proximal (*j*_*r*_=2.41 ± 0.25 × 10^–5^ cm s^−1^) segments of crown roots was much higher than the radial flow into the seminal roots, which was below our detection limit (*j*_*r*_≤5.0 × 10^–8^ cm s^−1^). The radial flow into the lateral of crown was *j*_*r*_=8.68 ± 0.35 × 10^–6^ cm s^−1^. We did not observe any transport of D_2_O into the laterals of seminal roots.

### Axial hydraulic conductivity

**Fig. 6.**
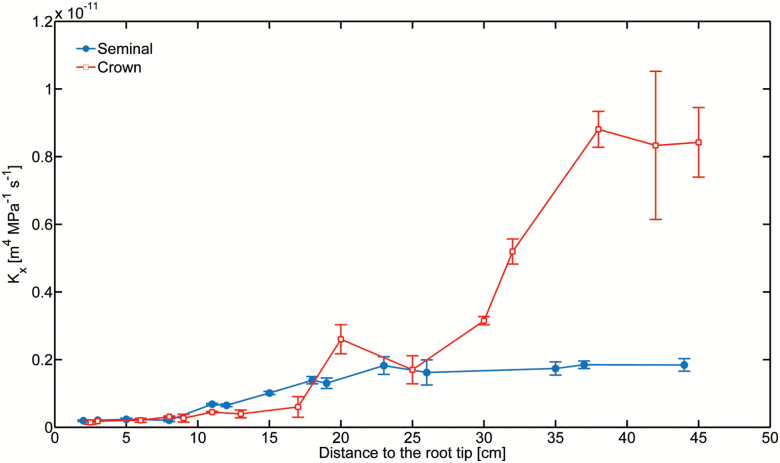
The axial hydraulic conductivity of seminal and crown roots in 5-week-old maize. The conductivities were similar in the most distal (younger) segments. Crown roots started to show higher conductivity at a distance of 18–30 cm from the root tip. Further away from the root tip (distance >30 cm), the crown roots were increasingly more conductive than the seminals. Data represent the mean ±SD (n=7). (This figure is available in color at *JXB* online.)

The axial hydraulic conductivity of seminal and crown roots was similar in the most distal (younger) segments. Crown roots started to show higher conductivity at a distance of 18–30 cm from the root tip. Further away from the root tip (distance >30 cm), the crown roots were increasingly more conductive than the seminal roots (Fig. 6).

## Discussion

We found that water was mainly taken up by crown roots and their laterals. Laterals of seminal roots, which were the main location of water uptake in younger plants (Ahmed *et al.*, 2016), took up a negligible amount of water. In contrast to seminal roots, crown roots were also able to take up water from their distal segments. These results are summarized in Fig. 5.

Note that the results are likely to be affected by some model assumptions. For instance, treating the root tissue as a homogeneous medium and assuming that the diffusion coefficient of the root tissues did not change between day and night are strong simplifications. Such approximations are justified by the need to reduce the number of unknown parameters in the inverse problem, but they bring some error to the estimation of the fluxes and the diffusion coefficients. For instance, root permeability has been shown to vary by 2- to 3-fold between day and night (Parsons and Kramer, 1974; Javot and Maurel, 2002). Such changes in permeability are associated with diurnal fluctuations in aquaporin activity influencing the radial water flow through the roots (Bramley *et al.*, 2009; Chaumont and Tyerman, 2014; Caldeira *et al.*, 2014). However, it is unlikely that such errors explain the different order of magnitude of the water fluxes between crown and seminal roots. Therefore, the conclusion that crown roots take up more water than seminal roots in mature maize is solid.

The hypothesis that water uptake in mature maize is dominated by crown (nodal) roots while seminal roots and their laterals stop taking up water was debated in the past, without a general consensus being reached. In fact, many studies demonstrated that the embryonic primary and seminal roots together with their laterals function only temporarily, and gradually die off (especially the laterals of the seminals) when the crown (nodal) roots develop (often referred to as the permanent root system). Other studies, however, confuted this generalization for grasses and especially maize (McCully, 1995). The debate is far from being settled.

In maize, we believe that seminal roots and their laterals do not actually die off but rather their contribution to root water uptake becomes quite small. Many studies have been conducted to determine the contribution of primary and crown root systems to growth and yield (Rostamza *et al.*, 2013). For instance, it was shown that the seminal roots of maize supply one-fifth of the water used by the plant during its lifetime, and the amount of water supplied decreases after tassling and increases again during grain filling. Krassovsky (1926) compared the contribution of the primary and nodal roots to water uptake in barley, wheat, and rye, concluding that the primary root absorbed 25% of the water and nodal roots absorbed the remaining 75%. Similarly, Sallans (1942) found that nodal roots contributed to ~62% of the water for wheat yield. Shane and McCully (1999) compared maize with nodal roots with maize with amputated nodal roots. They found that despite the fact that the shoot looked similar, plants without nodals and with just a single primary root wilted at mid-day (i.e. at high vapor pressure deficit), which suggests that nodal roots are important to sustain a high transpiration rate. Our results are in line with these studies and provide clear evidence that crown (nodal) roots are more important in mature maize and together with their laterals represent the main location of water and possibly nutrient uptake.

In contrast to seminal roots, which do not take up water in the most distal 10–20 cm segments (Ahmed *et al.*, 2016), crown roots were also able to take up water from their most distal part. This would surely provide an advantage in arid regions and periods when water is mainly available in the subsoil and the transpiration demand is high.

The questions are: (i) why do crown roots take up much more water than the seminal roos; and (ii) why are crown roots, in contrast to seminal roots, able to take up water from their most distal parts? The answer to the first question comes in part from the axial conductivities. Differences in hydraulic conductivities between crown and seminal roots appear after ~15–20 cm from the root tip. The fact that crown roots are more axially conductive in the proximal segments favors the propagation of the tension toward the crown roots rather than toward the seminal roots. Consequently, the driving force needed to take up water is greater along the crown roots. An additional reason is that crown roots are connected to the vascular system of the shoot above the seminal roots and therefore divert the transmission of the tension away from the leaves toward the crown roots, almost isolating the seminal roots from the water flow. An architectural model of water flow in the roots including a preferential connection of the crown to the shoot is needed to confirm our concept.

The answer to the second question is that the higher hydraulic axial conductivity in the proximal segments combined with shorter and fewer laterals compared with seminal roots allow a more uniform water potential along the root. In fact, lower uptake from laterals leads to lower dissipation of tension along the roots and thus to a greater uptake in distal segments. As for the first question, an architectural model of root water uptake combined with detailed measurements of root hydraulic properties should be used to confirm our concept.

Our results can be reasonably generalized to other maize varieties. Indeed, the fact that crown roots have more and larger xylem vessels (which leads to a higher axial conductivity) has been observed in other varieties (Tai *et al.*, 2016). Additionally, the fact that crown roots are connected to the shoot above the seminal roots is a common (and rather trivial) property of crown roots. Concerning their branching, crown roots have been shown to have shorter laterals in comparison with seminal roots (Hochholdinger *et al.*, 2004). These observations combined with our mechanistic explanation support a reasonable generalization of our results.

In conclusion, we showed that crown roots have a different capacity to transport water compared with seminal roots. Acknowledging such differences between root types is crucial to understand optimal root traits for water extraction from the soil.

## Supplementary data

Supplementary data are available at *JXB* online.

Fig. S1. Illustration of deuterated water (D_2_O) transport into a root that is partially immersed in D_2_O.

Supplementary Figure S1

## References

[CIT0001] Ahmed MA, Zarebanadkouki M, Kaestner A, Carminati A 2016 Measurements of water uptake of maize roots: the key function of lateral roots. Plant and Soil 398, 59–77.

[CIT0002] Bishopp A, Lynch JP 2015 The hidden half of crop yields. Nature Plants 1, 15117. 27250548 10.1038/nplants.2015.117

[CIT0003] Bramley H, Turner NC, Turner DW, Tyerman SD 2009 Roles of morphology, anatomy, and aquaporins in determining contrasting hydraulic behavior of roots. Plant Physiology 150, 348–364. 19321713 10.1104/pp.108.134098 PMC2675714

[CIT0004] Caldeira CF, Jeanguenin L, Chaumont F, Tardieu F 2014 Circadian rhythms of hydraulic conductance and growth are enhanced by drought and improve plant performance. Nature Communications 5, 5365. 10.1038/ncomms6365 PMC4241992 25370944

[CIT0005] Chaumont F, Tyerman SD 2014 Aquaporins: highly regulated channels controlling plant water relations. Plant Physiology 164, 1600–1618. 24449709 10.1104/pp.113.233791 PMC3982727

[CIT0006] Da Ines O, Graf W, Franck KI, Albert A, Winkler JB, Scherb H, Stichler W, Schäffner AR 2010 Kinetic analyses of plant water relocation using deuterium as tracer—reduced water flux of Arabidopsis pip2 aquaporin knockout mutants. Plant Biology 12(Suppl 1), 129–139. 20712628 10.1111/j.1438-8677.2010.00385.x

[CIT0007] Doussan C, Pagès L, Vercambre G 1998 Modelling of the hydraulic architecture of root systems: an integrated approach to water absorption—model description. Annals of Botany 81, 213–223.

[CIT0008] Frensch J, Steudle E 1989 Axial and radial hydraulic resistance to roots of maize (*Zea mays* L.). Plant Physiology 91, 719–726. 16667092 10.1104/pp.91.2.719 PMC1062061

[CIT0009] Hochholdinger F 2009 The Mmaize root system: morphology, anatomy, and genetics. In: Bennetzen JL, Hake SC, eds. Handbook of maize: its biology. New York: Springer, 145–160.

[CIT0010] Hochholdinger F, Park WJ, Sauer M, Woll K 2004 From weeds to crops: genetic analysis of root development in cereals. Trends in Plant Science 9, 42–48. 14729218 10.1016/j.tplants.2003.11.003

[CIT0011] Javot H, Maurel C 2002 The role of aquaporins in root water uptake. Annals of Botany 90, 301–313. 12234142 10.1093/aob/mcf199 PMC4240399

[CIT0012] Krassovsky I 1926 Physiological activity of the seminal and nodal roots of crop plants. Soil Science 21, 307.

[CIT0013] Leitner D, Meunier F, Bodner G, Javaux M, Schnepf A 2014 Impact of contrasted maize root traits at flowering on water stress tolerance—a simulation study. Field Crops Research 165, 125–137.

[CIT0014] Liu B-B, Steudle E, Deng X-P, Zhang S-Q 2009 Root pressure probe can be used to measure the hydraulic properties of whole root systems of corn (*Zea mays* L.). Botanical Studies 50, 303–310.

[CIT0015] McCully M 1995 How do real roots work? (some new views of root structure). Plant Physiology 109, 1–6. 12228578 10.1104/pp.109.1.1 PMC157558

[CIT0016] Ordin L, Kramer PJ 1956 Permeability of *Vicia faba* root segments to water as measured by diffusion of deuterium hydroxide. Plant Physiology 31, 468–471. 16654925 10.1104/pp.31.6.468 PMC540832

[CIT0017] Parsons LR, Kramer PJ 1974 Diurnal cycling in root resistance to water movement. Physiologia Plantarum 30, 19–23.

[CIT0018] Robbins NE, Dinneny JR 2015 The divining root: moisture-driven responses of roots at the micro- and macro-scale. Journal of Experimental Botany 66, 2145–2154. 25617469 10.1093/jxb/eru496 PMC4817643

[CIT0019] Rostamza M, Richards RA, Watt M 2013 Response of millet and sorghum to a varying water supply around the primary and nodal roots. Annals of Botany 112, 439–446. 23749473 10.1093/aob/mct099 PMC3698390

[CIT0020] Sallans BJ 1942 The importance of various roots to the wheat plant. Scientific Agriculture 23, 17–26.

[CIT0021] Shane MW, McCully ME 1999 Root xylem embolisms: implications for water flow to the shoot in single-rooted maize plants. Australian Journal of Plant Physiology 26, 107–114.

[CIT0022] Steudle E 2000 Water uptake by plant roots: an integration of views. Plant and Soil 226, 45–56.

[CIT0023] Tai H, Lu X, Opitz N, Marcon C, Paschold A, Lithio A, Nettleton D, Hochholdinger F 2016 Transcriptomic and anatomical complexity of primary, seminal, and crown roots highlight root type-specific functional diversity in maize (*Zea mays* L.). Journal of Experimental Botany 67, 1123–1135. 26628518 10.1093/jxb/erv513 PMC4753849

[CIT0024] Varney GT, Canny MJ 1993 Rates of water uptake into the mature root system of maize plants. New Phytologist 123, 775–786.

[CIT0025] Wang X-L, Canny MJ, McCully ME 1991 The water status of the roots of soil-grown maize in relation to the maturity of their xylem. Physiologia Plantarum 82, 157–162.

[CIT0026] Warren JM, Bilheux H, Kang M, Voisin S, Cheng C-L, Horita J, Perfect E 2013 Neutron imaging reveals internal plant water dynamics. Plant and Soil 366, 683–693.

[CIT0027] Zarebanadkouki M, Kroener E, Kaestner A, Carminati A 2014 Visualization of root water uptake: quantification of deuterated water transport in roots using neutron radiography and numerical modeling. Plant Physiology 166, 487–499. 25189533 10.1104/pp.114.243212 PMC4213081

