# Supplementary material for: Root type matters: measurement of water uptake by seminal, crown, and lateral roots in maize
Source: J Exp Bot. 2018 Jan 3;69(5):1199–206. doi: 10.1093/jxb/erx439 (PMC6019006; doi:10.1093/jxb/erx439)
Supplement: Supplementary Figure S1 [file erx439_suppl_supplementary_figure_s1.pdf]

Supplementary data

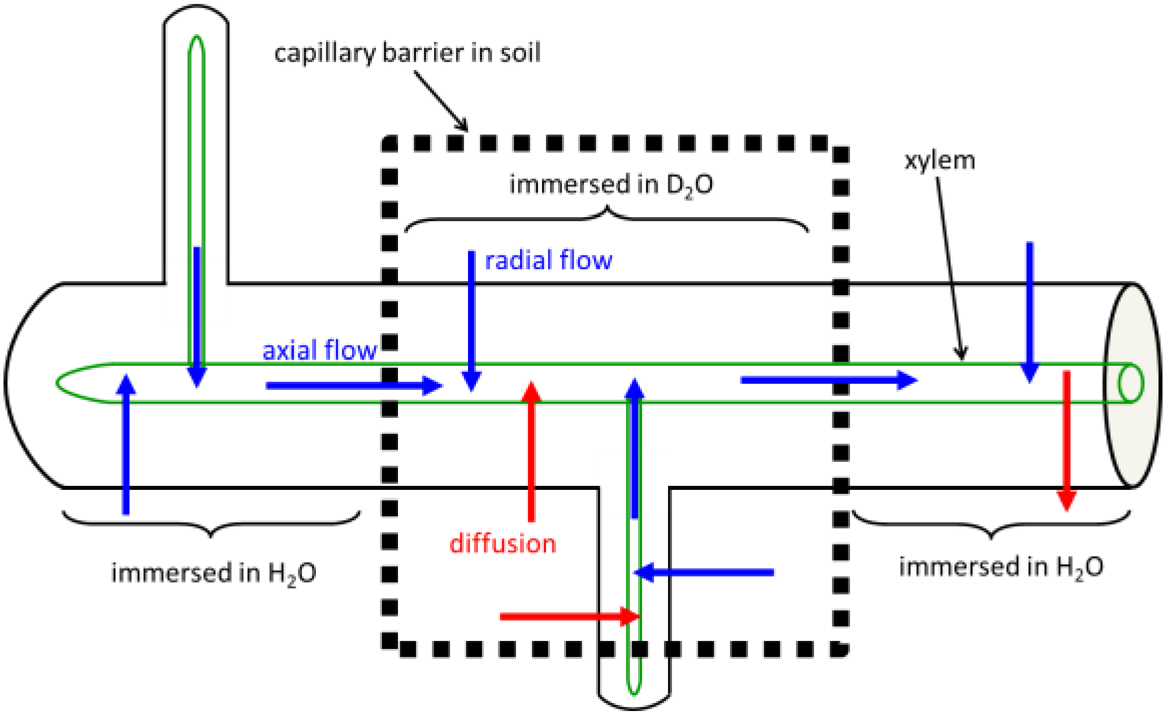

Fig. S1. Illustration of deuterated water (D_2O) transport into a root that is partially immersed in D_2O. The radial transport of D_2O into the root is driven by the concentration gradients between the soil and roots (diffusion, red arrows) and by convection following the transpiration stream (blue arrows). As D_2O reaches the xylem, it mixes with the incoming water flow and flows axially along the root. The capillary barriers were used to limit D_2O diffusion in soil.
